# Biallelic *LGI1* and *ADAM23* variants cause hippocampal epileptic encephalopathy via the LGI1–ADAM22/23 pathway

**DOI:** 10.1093/brain/awaf202

**Published:** 2025-06-02

**Authors:** Yoko Hirano, Yuri Miyazaki, Daisuke Ishikawa, Hiroki Inahashi, Zuhair Nasser Al-Hassnan, Giovanni Zifarelli, Peter Bauer, Javeria Raza Alvi, Tipu Sultan, Michelle L Thompson, Abdullah Sezer, Bahadır Konuşkan, Razan S Hajir, Ayman W El-Hattab, Stephanie Efthymiou, Ayuki Ishida, Norihiko Yokoi, Hans-Christian Kornau, Dietmar Schmitz, Harald Prüss, Henry Houlden, Yuji Ikegaya, Yuko Fukata, Masaki Fukata, Reza Maroofian

**Affiliations:** Department of Pediatrics, Graduate School of Medicine, The University of Tokyo, Tokyo 113-8655, Japan; Department of Pediatrics, The University of Tokyo Hospital, Tokyo 113-8655, Japan; Division of Neuropharmacology, Nagoya University Graduate School of Medicine, Nagoya, Aichi 466-8550, Japan; Graduate School of Pharmaceutical Sciences, The University of Tokyo, Tokyo 113-0033, Japan; Section of Mammalian Transgenesis, Center for Genetic Analysis of Behavior, National Institute for Physiological Sciences, National Institutes of Natural Sciences, Okazaki, Aichi 444-8787, Japan; College of Medicine, Alfaisal University, Department of Medical Genomics, Genomic Medicine Centre of Excellence, King Faisal Specialist Hospital & Research Centre, Riyadh 11211, Saudi Arabia; CENTOGENE GmbH, Rostock 18055, Germany; CENTOGENE GmbH, Rostock 18055, Germany; Department of Paediatric Neurology, The Children’s Hospital and the University of Child Health Sciences, Lahore, Punjab 54600, Pakistan; Department of Paediatric Neurology, The Children’s Hospital and the University of Child Health Sciences, Lahore, Punjab 54600, Pakistan; HudsonAlpha Institute for Biotechnology, Huntsville, AL 35806, USA; Department of Medical Genetics, Ankara Etlik City Hospital, Ankara 06170, Türkiye; Division of Pediatric Neurology, Department of Pediatrics, Ankara Etlik City Hospital, Ankara 06170, Türkiye; Research Institute for Medical and Health Sciences, University of Sharjah, Sharjah, United Arab Emirates; Department of Clinical Sciences, College of Medicine, University of Sharjah, Sharjah, United Arab Emirates; Clinical Genetics, University Hospital Sharjah, Sharjah, United Arab Emirates; Genetics and Rare Disease Center, Burjeel Medical City, Abu Dhabi, United Arab Emirates; Department of Neuromuscular Disorders, UCL Queen Square Institute of Neurology, University College London, London WC1N 3BG, UK; Division of Neuropharmacology, Nagoya University Graduate School of Medicine, Nagoya, Aichi 466-8550, Japan; Division of Neuropharmacology, Nagoya University Graduate School of Medicine, Nagoya, Aichi 466-8550, Japan; German Center for Neurodegenerative Diseases (DZNE) Berlin, Berlin 10117, Germany; Neuroscience Research Center (NWFZ), Cluster NeuroCure, Charité-Universitätsmedizin Berlin, Freie Universität Berlin and Humboldt-Universität zu Berlin, Berlin 10117, Germany; German Center for Neurodegenerative Diseases (DZNE) Berlin, Berlin 10117, Germany; Neuroscience Research Center (NWFZ), Cluster NeuroCure, Charité-Universitätsmedizin Berlin, Freie Universität Berlin and Humboldt-Universität zu Berlin, Berlin 10117, Germany; German Center for Neurodegenerative Diseases (DZNE) Berlin, Berlin 10117, Germany; Helmholtz Innovation Lab BaoBab (Brain Antibody-Omics and B-Cell Lab), Berlin 10117, Germany; Department of Neurology and Experimental Neurology, Charité-Universitätsmedizin Berlin, Freie Universität Berlin and Humboldt-Universität zu Berlin, Berlin 10117, Germany; Department of Neuromuscular Disorders, UCL Queen Square Institute of Neurology, University College London, London WC1N 3BG, UK; Graduate School of Pharmaceutical Sciences, The University of Tokyo, Tokyo 113-0033, Japan; Center for Information and Neural Networks, National Institute of Information and Communications Technology, Suita City, Osaka 565-0871, Japan; Institute for AI and Beyond, The University of Tokyo, Tokyo 113-0033, Japan; Division of Molecular and Cellular Pharmacology, Nagoya University Graduate School of Medicine, Nagoya, Aichi 466-8550, Japan; Division of Neuropharmacology, Nagoya University Graduate School of Medicine, Nagoya, Aichi 466-8550, Japan; Center for One Medicine Innovative Translational Research (COMIT), Nagoya University, Nagoya, Aichi 464-8601, Japan; Department of Neuromuscular Disorders, UCL Queen Square Institute of Neurology, University College London, London WC1N 3BG, UK

**Keywords:** LGI1, ADAM22, ADAM23, MAGUK, drug-resistant seizures, developmental and epileptic encephalopathy

## Abstract

Monoallelic pathogenic variants in *LGI1* cause autosomal dominant epilepsy with auditory features with onset in childhood/adolescence. LGI1 is a secreted neuronal protein, functions as a ligand for ADAM22/23, and regulates excitatory synaptic transmission and neuronal excitability in the brain. While biallelic *ADAM22* variants cause developmental and epileptic encephalopathy (DEE), the whole picture of LGI1–ADAM22/23 pathway-related diseases remains incompletely understood.

Through international genetic data sharing, we identified the first ultra-rare biallelic *LGI1* variants in six individuals from four consanguineous families. Affected individuals presented DEE with neonatal/infantile-onset epilepsy (*n* = 6/6), global developmental delay/intellectual disability (*n* = 6/6) and infant/premature death (*n* = 5/6). Brain MRI showed mild cerebral atrophy in a subset of patients (*n* = 3/6).

Functional analyses revealed that all *LGI1* variants result in reduced secretion and ADAM22-binding. Residual LGI1 function levels correlated with clinical severity, ranging from infantile lethality to intermediate phenotypes. Further, we observed epileptic discharges from the isolated whole hippocampus of *Lgi1*^–/–^ knockout mice, experimentally modelling the hippocampal origin of *LGI1*-related epilepsy. Automated behavioural analysis of a mouse model for *ADAM22*-related DEE revealed its impaired cognitive function. Furthermore, we report the first *ADAM23* variant associated with lethal neonatal-onset epilepsy and myopathy. Collectively, this study defines the LGI1–ADAM22/23 pathway-related disease spectrum.

## Teaser

Hirano *et al.* define a novel neurological disease spectrum involving the LGI1–ADAM22/23 pathway, based on the identification of ultra-rare biallelic *LGI1* variants associated with progressive developmental and epileptic encephalopathy, and a biallelic *ADAM23* variant associated with lethal neonatal-onset epilepsy.

## Introduction

Monoallelic pathogenic variants in *LGI1*, a secreted neuronal protein, cause autosomal dominant epilepsy with auditory features [ADEAF, previously known as autosomal dominant lateral temporal lobe epilepsy (ADLTE), OMIM: #600512)].^1,2^ ADEAF is characterized by focal epilepsy of childhood/adolescence with auditory auras. LGI1 functions as a ligand for ADAM22 and ADAM23 transmembrane proteins.^3,4^ ADAM22/23 are catalytically inactive ADAM family members and highly expressed in the brain. ADAM22 directly binds to PSD-95 family membrane-associated guanylate kinases (MAGUKs) through its C-terminal PDZ-binding motif. LGI1 and ADAM22/23 form the 2:2 heterotetrameric complex and mediates trans-synaptic protein networks, including MAGUKs (e.g. postsynaptic Dlg4/PSD-95, presynaptic CASK, etc.) and functional transmembrane proteins, such as ionotropic glutamate receptors and voltage-dependent potassium channels (K_v_1 channels) (Supplementary Fig. 1).^5,6^ Thus, the LGI1–ADAM22/23–MAGUK supercomplex finely regulates excitatory synaptic transmission, synaptic plasticity and neuronal excitability.^4,5,7^

Genetic analyses support the biological importance of the LGI1–ADAM22/23–MAGUK pathway. Mice lacking *Lgi1*, *Adam22* or *Adam23* commonly display lethal epilepsy in infancy.^4,8-10^ Heterozygous *Lgi1* knockout (KO), *Lgi1*^+/–^ mice show no spontaneous seizures, but increased seizure susceptibility to the convulsant pentylenetetrazole.^4^ The modest phenotype of *Lgi1*^+/−^ mice is consistent with the incomplete penetrance (∼70%) of monoallelic ADEAF variants of *LGI1*, which cause the functional loss of LGI1 (i.e. secretion or ADAM22 binding deficiency).^11,12^ In addition, autoantibodies to LGI1 cause autoimmune-mediated limbic encephalitis (LE), characterized with subacute onset of amnesia, seizures and cognitive dysfunction in adulthood.^13-15^ Because LGI1 autoantibodies inhibit the interaction between LGI1–ADAM22^15^  ^,16^ and/or internalize the LGI1–ADAM22 complex,^17^ loss or reduction of the LGI1–ADAM22/23 complex, by either genetic or non-genetic reasons, causes epileptic disorders. Despite a variety of neurological symptoms in LE, no *LGI1* variants related to neurological symptoms besides pure epilepsy have been reported.

Recently, biallelic pathogenic variants in *ADAM22* were reported in patients with neonatal/infantile-onset developmental and epileptic encephalopathy (DEE).^18^ The patients show more severe and wider ranges of clinical phenotypes including developmental/intellectual delay and behavioural abnormalities than those with *LGI1*-related ADEAF. Very recently, a population-wide genomic study of 748 879 individuals identified a novel association of *ADAM23* with epilepsy as *LGI1*.^19^ No pathogenic variants in *ADAM23*, however, have been reported. Pathogenic variants in MAGUKs (*DLG4*/*PSD-95*, *CASK*, etc.) have been reported in patients with DEE.^20^ Thus, the significance of the LGI1–ADAM22/23–MAGUK pathway in human brain functions and diseases remains unclear.

Here, we identified the first biallelic variants in *LGI1* associated with DEE and in *ADAM23* with neonatal-onset epilepsy. Our functional analysis revealed the genotype-function-phenotype correlations, providing insights into the clinical spectrum for *LGI1*–*ADAM22/23*-related brain disorders.

## Materials and methods

For details on the patient identification, clinical assessments, genetic investigations, *in vitro* functional studies, *in toto* electrophysiological experiments, behavioural analysis using the IntelliCage system and statistical analyses, refer to the online Supplementary material, ‘Materials and methods’ section.

## Results

### Clinical features

**Figure 1:**
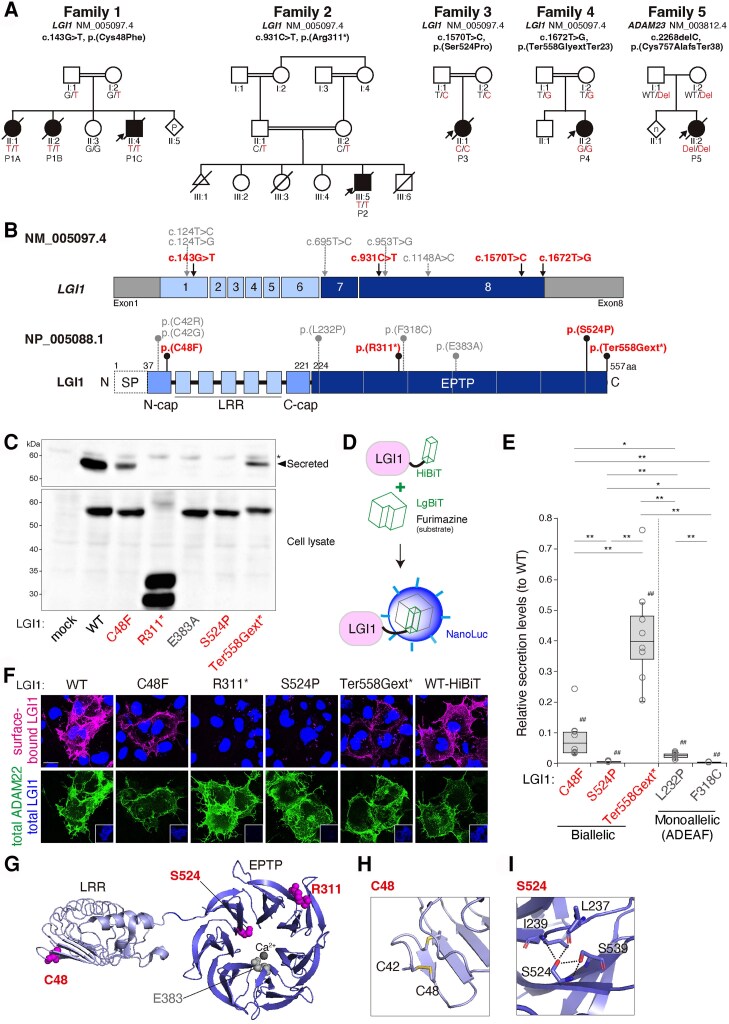
**Pedigrees and functional analyses for biallelic *LGI1* and *ADAM23* variants.** (**A**) Pedigrees of seven individuals from five families with segregating biallelic *LGI1* (Families 1–4) or *ADAM23* (Family 5) variants are shown. (**B**) Schematic diagram of *LGI1* gene and protein. LGI1 consists of the N-terminal leucine-rich repeat (LRR, light blue) and the C-terminal epitempin-repeat (EPTP, dark blue) domains. The N-terminal secretion signal peptide (SP, enclosed by dotted lines) is removed in the secreted LGI1. The RefSeq ID NM_005097.4 is used to indicate all variants. Grey = reported monoallelic autosomal dominant epilepsy with auditory features (ADEAF) variants; red = new biallelic variants (**B**, **C**, **E** and **G**). Ter558Gext* means Ter558GlyextTer23 (**B**, **C**, **E** and **F**). (**C**) Secretion test for LGI1 variants. Shown are western blots, with anti-LGI1 antibody, of conditioned medium from transfected HEK293T cells (*top*) and cell lysates (*bottom*). A closed arrowhead indicates the position of the secreted LGI1 protein. An asterisk indicates the non-specific signal. The lower band of R311 variant in the *bottom* panel seems to be its degradation product. The data shown are representative of four experiments. Glu383Ala is a reported monoallelic ADEAF variant. WT = wild-type. (**D**) Principle of the HiBiT system. When a HiBiT-tagged LGI1 secreted in the culture medium interacts with a cell-impermeable LgBiT, they form an active nanoluciferase to produce luminescent signals in the presence of its substrate, furimazine. (**E**) The HiBiT system shows that secretion levels of LGI1 variants are variably reduced compared with WT. *P*-values were determined by Kruskal-Wallis test: ^##^*P* < 0.01 (versus WT, Steel test); *P < 0.05, **P < 0.01 (between variants, Steel-Dwass test); *n* = 8. Monoallelic variants in ADEAF: Leu232Pro, with partial secretion and incomplete penetrance; Phe318Cys, no secretion and complete penetrance.^12^ (**F**) The ADAM22-binding of LGI1 variants. Indicated cDNAs of *LGI1* variants and *ADAM22* were co-expressed into COS-7 cells, and cell-surface bound LGI1 through ADAM22 was live-labelled by anti-LGI1 antibody (magenta). After fixation and permeabilization of cells, protein expression of ADAM22 (green, total) and LGI1 [blue (pseudocolour) in *insets*, total] was validated. Nuclear DNA was stained by Hoechst 33342 (blue) to distinguish transfected from untransfected cells (*top*). HiBiT-tagged LGI1 WT (Fig. 1D) showed the intact ADAM22 binding. Scale bar = 20 μm. (**G**–**I**) Mapping of three biallelic *LGI1* variants on the LGI1 structure (extracted from PDB #5Y31). The EPTP β-propeller domain (**G**) is responsible for the ADAM22 binding. The corresponding amino acid residues are shown as magenta or light grey spheres. Close-up views of Cys48 (**H**) and Ser524 (**I**) are shown.

The clinical features available of six patients (Patients P1A–C, P2, P3 and P4) with *LGI1* variants identified from four consanguineous families are described in Fig. 1A, Supplementary material and Supplementary Table 1, and summarized in Table 1. All affected individuals presented with neonatal/infantile-onset seizures (0–4 months) that were drug-resistant. Most affected individuals had generalized tonic-clonic seizures (*n* = 5/6). All affected individuals displayed mild-to-profound intellectual disability with delayed motor milestone attainment and remained non-verbal except for one affected individual (Patient P4). Only two of six affected individuals were able to sit and walk independently (Patients P1A and P4). During clinical examination, all affected individuals manifested profound general hypotonia in the first years of life, and four of five exhibited spastic features. No consistent extra-neurological manifestations or dysmorphic features were noted. Patient P2 died at the age of 9 months due to status epilepticus and developed aspiration pneumonia. Patient P3 suddenly died of unknown causes at the age of 4 years. Three patients (Patients P1A, P1B and P1C) died of respiratory failure at the age of 24, 21 and 11 years, respectively. Thus, five of the six patients with biallelic *LGI1* variants died young.

**Table 1:** Clinical characteristics of seven affected individuals with pathogenic variants in *LGI1* or *ADAM23* and available clinical data

	Patient P1A	Patient P1B	Patient P1C	Patient P2	Patient P3	Patient P4	Patient P5
Age^a^ (years)	24*	21*	11*	0.75*	4*	3.75	0.17*
Gender	Female	Female	Male	Male	Male	Female	Female
Ethnicity	Saudi Arabian	Saudi Arabian	Saudi Arabian	Pakistani	Jordanian	Turkish	African American
Consanguinity of parents	Yes	Yes	Yes	Yes	Yes (double cousins)	Yes	Unknown
Gene	*LGI1*	*LGI1*	*LGI1*	*LGI1*	*LGI1*	*LGI1*	*ADAM23*
cDNA variant^b^	c.143G>T	c.143G>T	c.143G>T	c.931C>T	c.1570T>C	c.1672T>G	c.2268delC
Protein variant	p.(Cys48Phe)	p.(Cys48Phe)	p.(Cys48Phe)	p.(Arg311*)	p.(Ser524Pro)	p.(Ter558GlyextTer23)	p.(Cys757Alafs*38)
Sequence method	WES, Sanger (trio)	WES, Sanger (trio)	WES, Sanger (trio)	WES, Sanger (trio)	WES (trio)	WES, Sanger (trio)	WGS, Sanger (trio)
Seizure onset (months)	0	0	0	0	0	4	0
Seizure type	Tonic-clonic	Tonic-clonic	Tonic-clonic	Tonic-clonic	Tonic-clonic	Focal	Tonic, myoclonic
Drug-resistant	Yes	Yes	Yes	Yes	Yes	Yes ∼ No (at present)	Unknown
EEG	Slowed background activity	NA	Multi-focal epileptic discharges, slowed background activity	Focal epileptic discharges > generalized epileptic discharges	Multi-focal epileptic discharges	Focal epileptic discharges	Slowed background activity
Motor delay	Yes	Yes	Yes	Yes	Yes	Yes	Never attained
Walks unsupported (age)	Yes (unknown)	Never attained	Never attained	Never attained	Never attained	Yes (20 months)	Never attained
Neurology	NA	Hypert, spasticity, muscle atrophy	Hypert, spasticity, muscle atrophy	Hypert, spasticity, muscle atrophy	Hypot (axial)/Hypert (peripheral), spasticity	Normal	Hypert, limb contractures
Verbal speech (age)	Never attained	Never attained	Never attained	Never attained	Never attained	Yes (simple words at 2.5 years, simple sentences at 3 years)	Never attained
GDD/ID	Severe	Severe	Severe	Severe	Severe	Mild	Never attained
Brain MRI (age)	Cerebral atrophy (8 years)	Cerebral atrophy, hypomyelination, delayed myelination (2 years); normal (4 years)	Normal (4 months)	Normal (3 months)	Normal (2 months)	Cerebral atrophy, corpus callosum thinning, ventriculomegaly (11 months)	Normal (unknown)

GDD = global developmental delay; Hypert = hypertonia; Hypot = hypotonia; ID = intellectual disability; NA = not available; WES = whole exome sequencing; WGS = whole genome sequencing.

^a^Age at last evaluation (the age of death is indicated with an asterisk).

^b^RefSeq IDs NM_005097.4 and NM_003812.4 are used for *LGI1* and *ADAM23*, respectively.

Brain MRI revealed mild cerebral atrophy in three of six subjects (Patients P1A, P1B and P4). One subject presented with hypomyelination of the frontal lobes and the corpus callosum and delayed myelination of the temporal lobes (Patient P1B). Corpus callosum thinning and ventriculomegaly were noted in one subject (Patient P4, Supplementary Fig. 2).

Additionally, we identified one patient (Patient P5) with the first biallelic variant in *ADAM23* (Fig. 1A, Supplementary material, Supplementary Table 1 and summarized in Table 1). Patient P5 presented with neonatal-onset seizures characterized by tonic or myoclonic seizures. EEG showed excess delta slowing in the background. Brain MRI was reported as normal by radiology; however, the treating paediatric neurologists raised suspicion for pachygyria. In addition, Patient P5 manifested limb hypertonia, limb joint contractures and the absence of deep tendon reflexes and primitive reflexes. The muscle biopsy showed severe myopathic changes. Patient P5 died of respiratory failure at the age of 2 months.

### Genetic findings

Whole-exome sequencing or whole-genome sequencing was performed in all families. The results are summarized in Fig. 1B, Table 1, Supplementary Tables 1 and 2 and described in detail in the Supplementary material. Briefly, biallelic variants in *LGI1* were identified in six affected individuals, which segregated within the families (Families 1–4) (Fig. 1A). All families’ parents of affected individuals were consanguineous. Variants identified include: missense variants c.143G>T; p.(Cys48Phe) (Patients P1A, P1B and P1C) and c.1570T>C; p.(Ser524Pro) (Patient P3); a nonsense variant c.931C>T; p.(Arg311*) (Patient P2); and a no-stop change variant c.1672T>G; p.(Ter558GlyextTer23) (Patient P4). All identified variants are absent in biallelic states in large genomic databases, and were predicted to be deleterious by most *in silico* prediction tools, except for c.1672T>G; p.(Ter558GlyextTer23) (Patient P4) (Supplementary Table 2). The c.931C>T variant transcript has a premature stop codon [p.(Arg311*)] and results in a truncated LGI1 with the EPTP domain disrupted (Fig. 1B).

The biallelic *ADAM23* frameshift variant [c.2268delC; p.(Cys757Alafs*38)] was identified within one family (Family 5) (Fig. 1A). The c.2268delC variant transcript containing a premature stop codon is likely to undergo nonsense-mediated decay. Even if expressed, the resultant ADAM23 protein p.(Cys757Alafs*38) lacks the transmembrane domain and could function as the loss-of-function variant. No other pathogenic/likely pathogenic variants were identified in other relevant disease-causing genes. Interestingly, the *ADAM23* locus was within loss of heterozygosity with 12.7 Mb at chromosome 2q32.3-q33.3, which is associated with a lot of different syndromes (OMIM: #612313).

### Functional characterization

To investigate the functional consequences of the *LGI1* variants on their secretion, we expressed wild-type LGI1 (WT) or newly identified LGI1 variants in HEK293T cells. Western blotting showed that LGI1 WT was efficiently secreted as previously reported (Fig. 1C).^12^ In contrast, secretion levels of LGI1 variants Cys48Phe and Ter558GlyextTer23 were reduced but partially preserved, and truncated Arg311* and Ser524Pro were hardly secreted (for the R311 variant; also see Supplementary Fig. 3, conditioned medium), showing the similar defect levels to that of the previously characterized Glu383Ala ADEAF variant.^12^ To more sensitively and precisely quantify the secretion levels of LGI1 proteins, we took advantage of the HiBiT extracellular system, based on the split nanoluciferase complementation assay (Fig. 1D). Tight binding of an 11-amino acid HiBiT peptide to an inactive large subunit of the enzyme (LgBiT) produces an active nanoluciferase. Here, the amount of secreted HiBiT-tagged LGI1 (LGI1-HiBiT) was measured by adding the cell-impermeable LgBiT and a luminescent substrate furimazine to the culture medium. We found differently reduced secretion levels of Cys48Phe, Ser524Pro and Ter558GlyextTer23 variants ∼6.7%, 0.5% and 40% (median, compared to LGI1 WT), respectively (Fig. 1E). Monoallelic ADEAF variants previously classified as the secretion-defective variants,^12^ p.Leu232Pro and p.Phe318Cys (Fig. 1E) together with p.Glu383Ala (Fig. 1C), caused consistently large secretion defects (0%–3%).

Next, to examine whether the secreted LGI1 variants bind to ADAM22 on the cell-surface, we performed the cell-surface binding assay. When LGI1 WT was co-expressed with ADAM22 in COS-7 cells, LGI1 efficiently bound to ADAM22 on the cell surface as previously reported (Fig. 1F).^18,21^ Importantly, Cys48Phe and Ter558GlyextTer23 variants showed the cell-surface binding, whereas Arg311* and Ser524Pro did not show any binding, indicating that secreted Cys48Phe and Ter558GlyextTer23 variants serve as functional ligands for ADAM22. Quantification using the HiBiT system showed that all the variants tested showed significant reduction in the net binding to ADAM22-expressing cells compared to LGI1 WT (Supplementary Fig. 4A). Cys48Phe and Ter558GlyextTer23 showed the similar levels of ADAM22 binding and significantly more binding than non-secreted Ser524Pro. Given the better secretion of Ter558GlyextTer23 than Cys48Phe (Fig. 1E), it is importantly suggested that Cys48Phe variant retains the intact binding activity to ADAM22 whereas Ter558GlyextTer23 shows the partial defect in ADAM22 binding. Then, we asked whether 4-phenylbutyrate (4PBA), which is a chemical chaperone and corrects the misfolding of several LGI1 ADEAF variants^12^ and ΔF508-CFTR,^22^ could increase the secretion levels of these LGI1 variants. We generated HEK293T cell lines stably expressing LGI1 WT or variants Cys48Phe, Ser524Pro, or Ter558GlyextTer23 and cultured in the presence or absence of 4PBA. We found that treatment with 4PBA (5 mM) for 72 h significantly increased the secretion of LGI1 Cys48Phe as well as WT, whereas it reduced the secretion of Ser524Pro and Ter558GlyextTer23 (Supplementary Fig. 4B). These results indicate that (i) p.Arg311* is functionally null; (ii) p.Ser524Pro is non-secretable; (iii) p.Cys48Phe and p.Ter558GlyextTer23 are partially secretable and bind to ADAM22; and (iv) 4PBA treatment differently affects the secretion levels of LGI1 variants, and increases the secretion of Cys48Phe.

Different effects of three LGI1 variants (p.Cys48Phe, p.Ser524Pro and p.Ter558GlyextTer23) on their secretion and ADAM22-binding could be explained based on the protein structure of the LGI1–ADAM22 complex (PDB #5Y31; Fig. 1G–I). The p.(Cys48Phe) disrupts an intramolecular disulfide bond between Cys42 and Cys48 in the N-terminal cap of leucine-rich repeat (LRR) (Fig. 1H). Cys42 variants [p.(Cys42Arg), p.(Cys42Gly)] (Fig. 1B) were reported in ADEAF patients and similarly cause the secretion defect.^6,12^ The p.(Ser524Pro) substitution disrupts three hydrogen bonds between Ser524 and Leu237/Ile239/Ser539, largely destabilizing the EPTP domain (Fig. 1I). A no-stop variant p.(Ter558GlyextTer23) adds the extra 23 amino acids to the C-terminus of LGI1 and might slightly disturb the EPTP domain structure to cause modest defects in secretion and ADAM22 binding.

**Figure 2:**
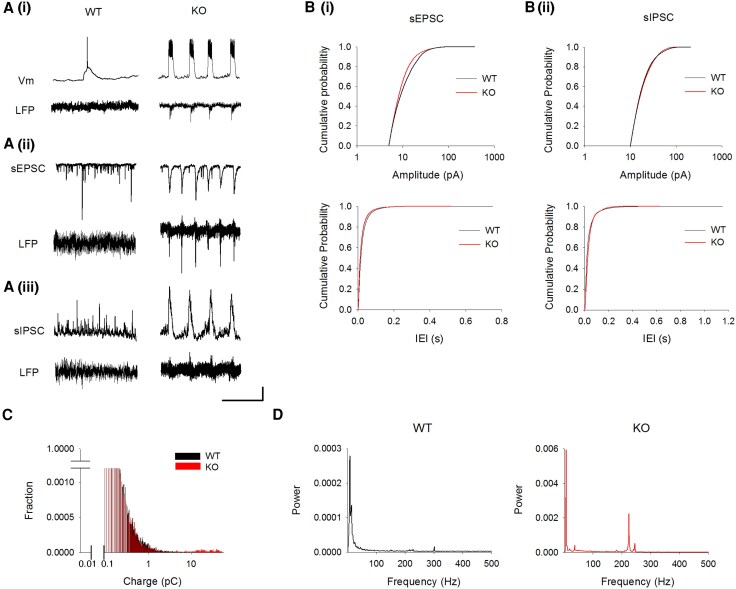
***In toto* whole-cell patch-clamp and LFP recordings from hippocampal CA3 pyramidal neurons and CA3 stratum pyramidale of *Lgi1*** ^**−/−**^  **mice, respectively**. (**A**) Representative traces of membrane potentials (Vm) and local field potentials LFPs [**A**(**i**)], spontaneous excitatory postsynaptic current (sEPSC) and LFPs [**A**(**ii**)], spontaneous inhibitory postsynaptic current (sIPSC) and LFPs [**A**(**iii**)] recorded in the same neuron. Scale bars = 20 mV, 0.1 mV, 1 s [**A**(**i**)], 50 pA, 0.1 mV, 1 s [**A**(**ii**)], 100 pA, 0.1 mV, 1 s [**A**(**iii**)]. WT = wild-type; KO = *Lgi1*^−/−^ mouse. (**B**) Cumulative frequency distribution curve of sEPSC amplitude and inter-event interval (IEI) [**B**(**i**)], sIPSC amplitude and IEI [**B**(**ii**)] (WT, *n* = 6; KO, *n* = 9). There are no significant differences between them (Kolmogorov-Smirnov test, *P* > 0.1). (**C**) Quantification of sEPSC charge transfer per second (WT, *n* = 6; KO, *n* = 9). (**D**) Power spectrum of LFPs. While delta and theta oscillations were recorded from hippocampal CA3 in WT mice, gamma and fast ripple oscillations were additionally recorded from hippocampal CA3 in *Lgi1* KO mice. Postnatal 16- to 18-day-old mice were used.

We next analysed *Lgi1* homozygous KO (*Lgi1*^−/−^) mice,^4^ which could serve as the counterpart model of human patients with the most deleterious biallelic variants such as p.(Arg311*) and p.(Ser524Pro). Because LGI1 is highly expressed in the hippocampus,^15^ we examined whether the hippocampus is the epileptic focus of *LGI1*-related epilepsy. Here, we used *in toto* preparations for the intact whole hippocampus.^23^ We dissected out a whole hippocampus from the mouse brain without slicing, and conducted whole-cell patch recordings from the CA3 pyramidal neuron, and simultaneously recorded local field potential (LFP) from the CA3 pyramidal cell layer. In the current-clamp mode, a CA3 neuron from an *Lgi1*^−/−^ mouse showed frequent bursts, and these bursts coincided with epileptic discharges of the CA3 region [Fig. 2A(i)]. WT CA3 neurons did not show any bursts and discharges. Because this recording was obtained from a whole hippocampus without afferents from other brain regions, this suggests that epileptic discharges originate in the hippocampus in *Lgi1*^−/−^ mice. Spontaneous excitatory postsynaptic currents (sEPSCs) and spontaneous inhibitory postsynaptic currents (sIPSCs) were recorded under the voltage-clamp mode. We found that one CA3 neuron of an *Lgi1*^−/−^ mouse showed the typical barrage [Fig. 2A(ii and iii)], indicating that one neuron receives synchronized inputs from many neurons. The cumulative frequency distribution of amplitude and inter-event interval (IEI) in sEPSCs and sIPSCs showed no significant difference between WT and *Lgi1*^−/−^ mice (Fig. 2B). The intrinsic electrical properties of CA3 neurons, such as membrane capacitance, input resistance, resting membrane potential and membrane time constant (Tau) were not different between genotypes (Supplementary Table 3). Importantly, barrage events in sEPSCs were observed only in CA3 neurons of *Lgi1*^−/−^ mice (Supplementary Table 4). We next quantified the charge transfer of sEPSC every 1 s. We found that small charges of sEPSC (<10 pC) were observed in both *Lgi1*^−/−^ mice and WT mice, whereas larger charges (>10 pC) attributed to barraged inputs were observed only in neurons from *Lgi1*^−/−^ mice (Fig. 2C). Furthermore, the power spectrum of LFPs identified the high-frequency oscillations including gamma (20–80 Hz) and fast ripple oscillations (>200 Hz) in hippocampal CA3 of *Lgi1*^−/−^ mice, but not WT mice (Fig. 2D). These results suggest that the hippocampal network excitability is increased intrinsically in the absence of afferent inputs to the hippocampus in the *Lgi1*^−/−^ mouse (Supplementary material, ‘Discussion’ section).

### Behavioural analysis of *Adam22*^ΔC5/ΔC5^ knock-in mice

**Figure 3:**
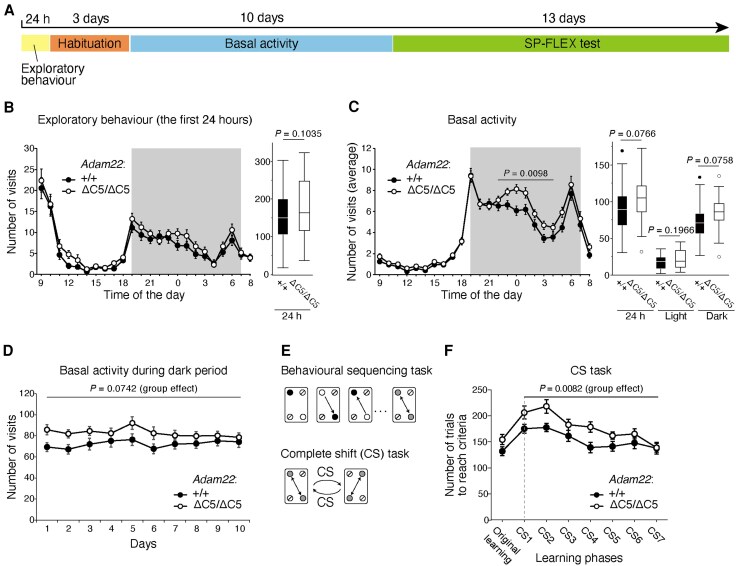
**Behavioural analysis of *Adam22*^ΔC5/ΔC5^ knock-in mice.** (**A**) Experimental schedule of behavioural analysis using IntelliCage. SP-FLEX test = self-paced behavioural sequencing learning and behavioural flexibility test. (**B**) Exploratory behaviour of *Adam22*^ΔC5/ΔC5^ (ΔC5/ΔC5) mice and wild-type (+/+) (WT) control mice. The total number of corner visits during the 24-h period of the exploratory behaviour task is shown in graph (**B**, *right*). Two-tailed Welch's *t*-test. *n* = 31 (WT mice) and *n* = 37 (*Adam22*^ΔC5/ΔC5^ mice). Mean ± standard error of the mean (SEM). (**C** and **D**) *Adam22*^ΔC5/ΔC5^ mice show high basal activity levels. (**C**) The number of corner visits during the middle phase of the dark period (22:00–04:00) was significantly higher in *Adam22*^ΔC5/ΔC5^ mice than in WT mice (**C**, *left*). The mean number of corner visits for the 24-h, 12-h light period, and 12-h dark period is shown in graph (**C**, *right*). Two-tailed Welch's *t*-test. Outliers are represented as closed (WT) and open (*Adam22*^ΔC5/ΔC5^) dots. (**D**) Higher basal activity levels during the dark phase were stably observed over 10 days in *Adam22*^ΔC5/ΔC5^ mice. Two-way repeated-measures ANOVA with *post hoc* Tukey-Kramer test. Group effect, *F*(1,65) = 3.293, *P* = 0.0742. *n* = 32 (WT mice) and *n* = 35 (*Adam22*^ΔC5/ΔC5^ mice). Mean ± SEM. (**E**) Behavioural sequencing task scheme for SP-FLEX test, showing opposite rewarded corners where the mouse has to go back and forth. In the complete shift (CS) task, the diagonally opposite rewarded corners are alternated sequentially with the other, upon achieving the successful visit-rate criterion at one diagonal. Black-filled circle = rewarded corner (active); open circle = rewarded corner (inactive); crossed-out circle = non-rewarded corner; grey-filled circles connected with double arrow = rewarding sequence. (**F**) *Adam22*^ΔC5/ΔC5^ mice have defects in learning and behavioural flexibility. The number of trials to reach the criteria was significantly higher in *Adam22*^ΔC5/ΔC5^ mice than in WT mice. Two-way repeated-measures ANOVA with *post hoc* Tukey-Kramer test. CS (CS1–7) group effect, *F*(1,61) = 7.475, *P* = 0.0082. *n* = 31 (WT mice) and *n* = 32 (*Adam22*^ΔC5/ΔC5^ mice). Mean ± SEM.

To investigate whether dysfunction of the LGI1–ADAM22/23 pathway affects the higher cognitive function, we used the *Adam22*^ΔC5/ΔC5^ mouse, which lacks the MAGUK interaction of ADAM22 and shows epilepsy at 2 to 8 months of age.^5^ This mouse model is a possible counterpart for human DEE cases with *ADAM22* truncation variants lacking the C-terminal PDZ binding motif and with milder clinical manifestations, delayed onset of epilepsy and distinct behavioural disorders.^18^ In contrast, homozygous KO mice of *Lgi1*, *Adam22* and *Adam23* show early lethal epilepsy (before postnatal 21 days), and thus are not suitable for behavioural analysis.^4,8-10^ We employed the IntelliCage system, which allows to test several behavioural tasks sequentially under the grouped-housing condition with the limited intervention of experimenters (Fig. 3A–F). Mice aged 8–11 weeks were introduced into the IntelliCage. There was no significant difference between WT and *Adam22*^ΔC5/ΔC5^ mice in exploratory behaviour (Fig. 3B). In contrast, *Adam22*^ΔC5/ΔC5^ mice exhibited an increased basal activity compared with WT mice, especially during the middle phase of the dark period, with a higher number of corner visits (Fig. 3C and D). To investigate the ability of learning and behavioural flexibility in *Adam22*^ΔC5/ΔC5^ mice, we used the self-paced behavioural flexibility (SP-FLEX) test,^24-26^ where the mice could obtain the reward (drinking water) by shuttling between two corners while adapting to its positional shifts (Fig. 3E). In the complete shift (CS) task, the rewarding corner pairs were positioned diagonally, and the positions alternated between the two diagonal patterns. We found that *Adam22*^ΔC5/ΔC5^ mice required more trials to reach the criteria than WT mice in the CS task (CS1–7, *P* = 0.0082) (Fig. 3F). These results revealed that *Adam22*^ΔC5/ΔC5^ mice showed impaired behavioural flexibility for learning rules that required complex behavioural changes. Collectively, the results revealed hyperactivity and reduced behavioural flexibility in *Adam22*^ΔC5/ΔC5^ mice, which are relevant for autism spectrum disorder, intellectual disability or attention-deficit/hyperactivity disorder. These symptoms are consistent with the phenotypes of *ADAM22*-related DEE. These results suggest that the LGI1–ADAM22–MAGUK pathway is associated with cognitive and behavioural disorders as well as epilepsy.

## Discussion

Monoallelic *LGI1* pathogenic variants are the most frequent cause of ADEAF, which is characterized by childhood/adolescence-onset focal epilepsy, typically without psychomotor retardation.^1,2^ Here, we describe the first biallelic *LGI1* and *ADAM23* pathogenic variants. Clinical features of patients with biallelic *LGI1* variants are substantially severe, showing early death and/or global developmental delay and intellectual disability besides epilepsy. Interestingly, very similar clinical features were observed in patients with biallelic *ADAM22*^18,21^ and *ADAM23* pathogenic variants (Supplementary Table 5). One patient with the *ADAM23* variant and 9 of 20 with the *ADAM22* variants^18,21^ died early in life. This is akin to mouse genetic evidence, in which *Lgi1*^−/−^, *Adam22*^−/−^ and *Adam23*^−/−^ mice commonly show severe, infantile lethal epilepsy.^4,8-10^ Together with phenotypic analyses of mouse models for *LGI1*- and *ADAM22*-variants, this study establishes the disease spectrum caused by the loss-of-function of the LGI1–ADAM22/23 pathway (Supplementary material, ‘Discussion’ section).

The clinical features among six affected individuals with biallelic *LGI1* variants vary ranging from death at 9 months old to survival at 24 years old. Our *in vitro* functional analysis of LGI1 variants revealed that residual LGI1 functions well-correlate with patients’ manifestation and survival. For example, Patient P2 with the truncated null variant p.(Arg311*) presented with the early onset epilepsy and died at 9 months old, whereas Patient P4 with the partially secretable variant p.(Ter558GlyextTer23) presented the later onset of epilepsy and showed clinical improvement with age. The milder course of Patient P4 compared to other cases is probably attributed to the higher level of variant secretion (Fig. 1E). Thus, our data broaden the clinical spectrum associated with *LGI1* variants, with various ages of onset and neurological involvements. The clinical features are mainly determined by (i) monoallelic or biallelic (dosage); and (ii) residual functional properties of individual *LGI1* variants (variant type) (Supplementary Table 6). It will be important to search for additional pathological variants of the LGI family and ADAM22 subfamily to define their specific roles in our brain. Consequently, this study depicts the varied spectrum of presentations of the LGI1-ADAM22/23-related diseases to facilitate the early evaluation, diagnosis and intervention.

## Supplementary Material

awaf202_Supplementary_Data

## Data Availability

De-identified clinical and genetic data related to this study can be shared upon reasonable request. Interested researchers may contact the corresponding authors to discuss data access in accordance with participant consent and institutional guidelines. Functional data can be shared with any qualified investigators from the corresponding authors upon reasonable requests.

## References

[1] Gu W, Brodtkorb E, Steinlein OK. LGI1 is mutated in familial temporal lobe epilepsy characterized by aphasic seizures. Ann Neurol. 2002;52:364–367. 12205652 10.1002/ana.10280

[2] Kalachikov S, Evgrafov O, Ross B, et al Mutations in LGI1 cause autosomal-dominant partial epilepsy with auditory features. Nat Genet. 2002;30:335–341. 11810107 10.1038/ng832 PMC2606053

[3] Fukata Y, Adesnik H, Iwanaga T, et al Epilepsy-related ligand/receptor complex LGI1 and ADAM22 regulate synaptic transmission. Science. 2006;313:1792–1795. 16990550 10.1126/science.1129947

[4] Fukata Y, Lovero KL, Iwanaga T, et al Disruption of LGI1-linked synaptic complex causes abnormal synaptic transmission and epilepsy. Proc Natl Acad Sci U S A. 2010;107:3799–3804. 20133599 10.1073/pnas.0914537107 PMC2840530

[5] Fukata Y, Chen X, Chiken S, et al LGI1-ADAM22-MAGUK configures transsynaptic nanoalignment for synaptic transmission and epilepsy prevention. Proc Natl Acad Sci U S A. 2021;118:e2022580118. 33397806 10.1073/pnas.2022580118 PMC7826393

[6] Yamagata A, Miyazaki Y, Yokoi N, et al Structural basis of epilepsy-related ligand-receptor complex LGI1-ADAM22. Nat Commun. 2018;9:1546. 29670100 10.1038/s41467-018-03947-w PMC5906670

[7] Seagar M, Russier M, Caillard O, et al LGI1 tunes intrinsic excitability by regulating the density of axonal Kv1 channels. Proc Natl Acad Sci U S A. 2017;114:7719–7724. 28673977 10.1073/pnas.1618656114 PMC5530646

[8] Chabrol E, Navarro V, Provenzano G, et al Electroclinical characterization of epileptic seizures in leucine-rich, glioma-inactivated 1-deficient mice. Brain. 2010;133:2749–2762. 20659958 10.1093/brain/awq171 PMC2929330

[9] Owuor K, Harel NY, Englot DJ, Hisama F, Blumenfeld H, Strittmatter SM. LGI1-associated epilepsy through altered ADAM23-dependent neuronal morphology. Mol Cell Neurosci. 2009;42:448–457. 19796686 10.1016/j.mcn.2009.09.008 PMC2783222

[10] Sagane K, Hayakawa K, Kai J, et al Ataxia and peripheral nerve hypomyelination in ADAM22-deficient mice. BMC Neurosci. 2005;6:33. 15876356 10.1186/1471-2202-6-33 PMC1142324

[11] Rosanoff MJ, Ottman R. Penetrance of LGI1 mutations in autosomal dominant partial epilepsy with auditory features. Neurology. 2008;71:567–571. 18711109 10.1212/01.wnl.0000323926.77565.ee PMC2652575

[12] Yokoi N, Fukata Y, Kase D, et al Chemical corrector treatment ameliorates increased seizure susceptibility in a mouse model of familial epilepsy. Nat Med. 2015;21:19–26. 25485908 10.1038/nm.3759

[13] Irani SR, Alexander S, Waters P, et al Antibodies to Kv1 potassium channel-complex proteins leucine-rich, glioma inactivated 1 protein and contactin-associated protein-2 in limbic encephalitis, Morvan's syndrome and acquired neuromyotonia. Brain. 2010;133:2734–2748. 20663977 10.1093/brain/awq213 PMC2929337

[14] Lai M, Huijbers MG, Lancaster E, et al Investigation of LGI1 as the antigen in limbic encephalitis previously attributed to potassium channels: A case series. Lancet Neurol. 2010;9:776–785. 20580615 10.1016/S1474-4422(10)70137-X PMC3086669

[15] Ohkawa T, Fukata Y, Yamasaki M, et al Autoantibodies to epilepsy-related LGI1 in limbic encephalitis neutralize LGI1-ADAM22 interaction and reduce synaptic AMPA receptors. J Neurosci. 2013;33:18161–18174. 24227725 10.1523/JNEUROSCI.3506-13.2013 PMC3828467

[16] Kornau HC, Kreye J, Stumpf A, et al Human cerebrospinal fluid monoclonal LGI1 autoantibodies increase neuronal excitability. Ann Neurol. 2020;87:405–418. 31900946 10.1002/ana.25666

[17] Ramberger M, Berretta A, Tan JMM, et al Distinctive binding properties of human monoclonal LGI1 autoantibodies determine pathogenic mechanisms. Brain. 2020;143:1731–1745. 32437528 10.1093/brain/awaa104 PMC7296845

[18] van der Knoop MM, Maroofian R, Fukata Y, et al Biallelic ADAM22 pathogenic variants cause progressive encephalopathy and infantile-onset refractory epilepsy. Brain. 2022;145:2301–2312. 35373813 10.1093/brain/awac116 PMC9337806

[19] Lal JC, Leu C, Boßelmann CM, Ivaniuk A, Pérez-Palma E, Lal D. Gene-burden meta-analysis of 748,879 individuals identifies LGI1-ADAM23 protein complex association with epilepsy. medRxiv. [Preprint]. doi: 10.1101/2024.12.30.24319794 PMC13360984 42216960

[20] Lelieveld SH, Reijnders MR, Pfundt R, et al Meta-analysis of 2,104 trios provides support for 10 new genes for intellectual disability. Nat Neurosci. 2016;19:1194–1196. 27479843 10.1038/nn.4352

[21] Muona M, Fukata Y, Anttonen AK, et al Dysfunctional ADAM22 implicated in progressive encephalopathy with cortical atrophy and epilepsy. Neurol Genet. 2016;2:e46. 27066583 10.1212/NXG.0000000000000046 PMC4817901

[22] Rubenstein RC, Egan ME, Zeitlin PL. In vitro pharmacologic restoration of CFTR-mediated chloride transport with sodium 4-phenylbutyrate in cystic fibrosis epithelial cells containing delta F508-CFTR. J Clin Invest. 1997;100:2457–2465. 9366560 10.1172/JCI119788 PMC508446

[23] Ikegaya Y, Sasaki T, Ishikawa D, et al Interpyramid spike transmission stabilizes the sparseness of recurrent network activity. Cereb Cortex. 2013;23:293–304. 22314044 10.1093/cercor/bhs006

[24] Balan S, Iwayama Y, Ohnishi T, et al A loss-of-function variant in SUV39H2 identified in autism-spectrum disorder causes altered H3K9 trimethylation and dysregulation of protocadherin beta-cluster genes in the developing brain. Mol Psychiatry. 2021;26:7550–7559. 34262135 10.1038/s41380-021-01199-7

[25] Endo T, Maekawa F, Võikar V, et al Automated test of behavioral flexibility in mice using a behavioral sequencing task in IntelliCage. Behav Brain Res. 2011;221:172–181. 21377499 10.1016/j.bbr.2011.02.037

[26] Miyazaki Y, Otsuka T, Yamagata Y, et al Oligodendrocyte-derived LGI3 and its receptor ADAM23 organize juxtaparanodal Kv1 channel clustering for short-term synaptic plasticity. Cell Rep. 2024;43:113634. 38194969 10.1016/j.celrep.2023.113634 PMC10828548

